# In Vitro Cytotoxic Activity of *Clinacanthus nutans* Leaf Extracts Against HeLa Cells

**DOI:** 10.31557/APJCP.2019.20.2.601

**Published:** 2019

**Authors:** Nor Hasyimah Haron, Zaleha Md Toha, Rafedah Abas, Mohammad Razak Hamdan, Nizuwan Azman, Melati Khairuddean, Hasni Arsad

**Affiliations:** 1 *Advanced Medical and Dental Institute, Universiti Sains Malaysia, Bertam, 13200 Kepala Batas,*; 2 *Centre for Drug Research, *; 3 *School of Chemistry, Universiti Sains Malaysia, 11800 Minden, Penang, Malaysia.*

**Keywords:** Clinacanthus nutans, phytochemical constituents, antiproliferative, apoptosis

## Abstract

**Objective:**

This study was conducted to investigate the antiproliferative activity of extracts of *Clinacanthus nutans* leaves against human cervical cancer (HeLa) cells.

**Methods:**

*C. nutans* leaves were subjected to extraction using 80% methanol or water. The methanol extract was further extracted to obtain hexane, dichloromethane (DCM), and aqueous fractions. The antiproliferative activity of the extracts against HeLa cells was determined. The most cytotoxic extract was furthered analyzed by apoptosis and cell cycle assays, and the phytochemical constituents were screened by gas chromatography-mass spectrometry (GC-MS).

**Results:**

All of the extracts were antiproliferative against HeLa cells, and the DCM fraction had the lowest IC_50 _value of 70 µg/mL at 48 h. Microscopic studies showed that HeLa cells exposed to the DCM fraction exhibited marked morphological features of apoptosis. The flow cytometry study also confirmed that the DCM fraction induced apoptosis in HeLa cells, with cell cycle arrest at the S phase. GC-MS analysis revealed the presence of at least 28 compounds in the DCM fraction, most of which were fatty acids.

**Conclusion:**

The DCM fraction obtained using the extraction method described herein had a lower IC_50_ value than those reported in previous studies that characterized the anticancer activity of *C. nutans* against HeLa cells.

## Introduction

The second leading cause of death in the world is cancer, and it is estimated that cancer will contribute to 9.6 million deaths in 2018 (Bray et al., 2018). Numerous factors are involved in cancer development, including genetics, health, nutrition, personal habits, and the environment. Cervical cancer is the fourth most common cancer among women globally; about 570,000 new cases were reported in 2018, representing 6.6% of all female cancers. Currently, the recommended treatment programme for this cancer involves surgery, chemotherapy, radiation, or other methods (World Health Organization, 2018). Herbal medicine has been increasingly recognized as a useful complementary and alternative therapy for patients with cancer. Numerous clinical studies have reported the positive effects of herbal medicines when taken with conventional therapeutics, in particular in terms of survival rate, the immune system, and quality of life of cancer patients (Yin et al., 2013). 

*Clinacanthus nutans* (*Burm. F.*) Lindau (CN), a medicinal herb that belongs to the Acanthaceae family, is one such natural product that might be effective in treating cancers, including human cervical cancer. Its leaf extract was effective in treating genital herpes, one of the leading causes of cervical cancer, by shortening duration of infection and reducing its severity (Sakdarat et al., 2006). Results of in vitro studies showed that CN was virucidal against herpes simplex virus (Jayavasu et al., 1992) and varicella zoster virus (Thawaranantha et al., 1992). In Malaysia, Singapore, and Thailand, an herbal tea made from the CN leaf is a traditional herbal remedy used to treat cancer patients. Some patients claimed to have recovered due to consistent and abundant consumption of CN juice or tea (Poonthananiwatkul et al., 2015; Siew et al., 2014). 

Numerous in vitro studies have reported antiproliferative and cytotoxic activities of CN extracts. For example, Yong et al., (2013) reported that the chloroform extract of CN had the greatest antiproliferative effect against the human erythroleukemia K-562 cell line (IC_50_: 47.70 μg/mL) and the human Burkitt’s lymphoma Raji cell line (IC_50_: 47.31 μg/mL) but not against the human neuroblastoma IMR-32 cell line. Quah et al., (2017) found that the methanol extract of CN showed cytotoxic activity against Hep G2 and MDA-MB-231 cells (IC_50_s: 13.3 and 18.7 μg/mL, respectively) but not against A549, HT-29, MCF-7, and CRL 1739 cells. Additionally, extracts of different plant parts (e.g., leaf, bark, stem, and roots) may have different effects on cancer cells. Arullappan et al., (2014) reported that the petroleum ether extract from CN leaves was cytotoxic against human cervical cancer (HeLa) cells (IC_50_: 18 μg/mL) but the stem extract was not (IC_50_: >100 μg/mL). However, the ethyl acetate extract from the stem was cytotoxic (IC_50_: 20 μg/mL) when compared to that of the leaf (IC_50_: 92 μg/mL) against the same cells (Arullappan et al., 2014).

The anticancer effects of CN may be due to its active compounds, and these effects may be further influenced by the type of extraction, solvent, and plant part used. For example, Khoo et al., (2015) reported that the terpenoids and sulfur-containing glucosides present in extracts of dried CN leaves were the main components responsible for in vitro antioxidant and α-glucosidase inhibitory effects. Teshima et al., (1997) identified six known C-glycosyl flavones (vitexin, isovitexin, shaftoside, isomollupentin 7-O-ß-glucopyranoside, orientin, and iso-orientin) from the butanol and water-soluble portions of the methanol extract of CN stems and leaves. The presence of these compounds indicates that CN possesses important biological activities, including antimicrobial, hepatoprotective, antioxidant, anti-inflammatory, and anti-neoplastic properties. 

This study was conducted to evaluate the in vitro antiproliferative activity of different solvent extracts of CN leaves against HeLa cells, which represent a human cancer cell model, in order to compare the bioacitivities of different solvent extracts. The most cytotoxic fraction was further analyzed using apoptosis and cell cycle phase assays, which are commonly used to determine how effective herbal remedies are against cancer cells. The compounds in this fraction also were analyzed using gas chromatography-mass spectrometry (GC-MS).

## Materials and Methods

*Plant material*

Fresh aerial parts of CN were collected at the HERBagus Sdn. Bhd. farm located at Pongsu Seribu, Kepala Batas, Penang, Malaysia (5°29’47.9’’N 100°26’43.2’’E). Samples containing leaves, stem, and root were identified and authenticated by the School of Biological Sciences, Universiti Sains Malaysia (deposited voucher number 11536 for reference). The leaves were separated from the stems and washed thoroughly with tap water followed by distilled water. The leaves were then stored at –80 °C prior the extraction process.

*Preparation of CN extracts*

80% methanol and fractioned [hexane, dichloromethane (DCM), and aqueous] extracts.

**Figure 1:**
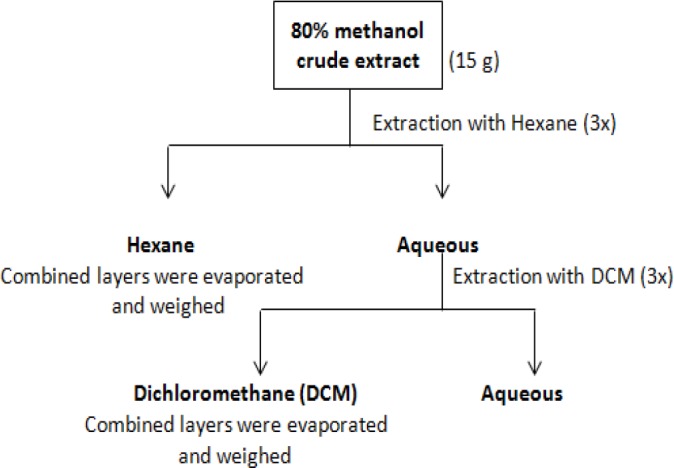
Flow Diagram Showing the Partitioning Extraction of the Aqueous Methanol Extract of CN by Liquid-Liquid Extraction

About 250 g of fresh CN leaves were blended into small pieces, soaked in 80% methanol for 24 h at room temperature, and filtered. The soaking process was repeated twice to ensure exhaustive extraction. The filtrate was concentrated in vacuo at 40°C to about 1/10 of the original volume and freeze-dried to obtain the 80% methanol crude extract. A large portion of this crude extract was partitioned with hexane (3 × 800 mL) and DCM (3 × 800 mL) by liquid-liquid extraction (Figure 1). These solvent fractions (hexane and DCM) together with the residual aqueous portion were concentrated to dryness in vacuo.

*Water extract*

About 500 g of fresh CN leaves were blended into small pieces, soaked in distilled water for 24 h at room temperature, and filtered. This process was repeated twice. The filtrate then was freeze-dried to obtain the crude extract.

*Cell line and culture*

HeLa cells (ATCC®CCL-2™) were purchased from the American Type Culture Collection (Manassas, VA, USA). Cells were cultured in Dulbecco’s modified eagle medium supplemented with 10% fetal bovine serum and 1% penicillin-streptomycin. Growth was maintained at 37°C in a humidified incubator containing 5% CO_2_. Cells were within 13 passages when the experiment was conducted.

*Antiproliferation assay*

All CN extracts were tested for antiproliferative activity against HeLa cells using the Sulforhodamine B (SRB) assay according to the previously described protocol (Skehan et al., 1998). Briefly, cells (2.5 x 10^4 ^cells/mL) were incubated in 96-well plates with a serial dilution of extracts starting with 4,000 µg/mL (water, 80% methanol, and its aqueous fraction) or 250 µg/mL (hexane and DCM fractions of the methanol extract) for 24, 48, and 72 h at 37°C with 5% CO_2_. The cells were fixed with 50 µL of 50% cold trichloroacetic acid for 30 min at room temperature followed by gentle tap water washing (5x) and then drying. Cells were then stained with 100 µl of 0.4% SRB in 1% acetic acid for 30 min, followed by washing with 1% acetic acid (4x). The plate was dried, 100 µL of 10 mM Tris buffer were added to each well, and the plate was shaken for 5 min. Relative cell viability was measured by scanning at 540 nm on a microplate reader (Bio-Tek® Instruments, Inc., Winooski, VT, USA). The cytotoxicity was determined using the previously described formula (Bendale et al., 2017):

Cytotoxicity (%) = Optical density of (sample – blank)/ Optical density of (control – blank) X 100

The inhibition concentration of extract that is able to inhibit cell proliferation by 50% (IC_50_) was calculated graphically from the cell proliferation curve. The extract at the time point with the lowest IC_50_ value was selected for further analysis. 

*Annexin V analysis of apoptosis*

Analysis of apoptosis in HeLa cells treated with the selected extract was performed using the Annexin V-FITC kit (Miltenyl Biotec GmbH, Bergisch Gladbach, Germany; Cat. No.: 130-092-052) via flow cytometry. HeLa cells (3.7 x 10^4^ cells/mL, 6 mL/flask) were incubated with three different doses (62.5, 125.0, and 250.0 µg/mL) of the selected extract for 48 h at 37°C with 5% CO_2_. All cells were harvested, including floating and adhering cells, after washing once with phosphate buffered saline (PBS). About 4 x 10^5^ cells were resuspended in 100 µl of binding buffer with 10 µl of Annexin V-FITC. The cells were washed with binding buffer after 15 min of incubation in the dark at room temperature, and 5 µl of propidium iodide solution then were immediately added prior to analysis by FACS Calibur flow cytometry (BD Biosciences, San Diego, CA, USA). A minimum of three independent replicates were analyzed using Mulltiset software. The percentages of viable, early apoptotic, late apoptotic, and necrotic cells were determined for a total of 10,000 events per sample.

*Morphological analysis*

Morphological changes of HeLa cells treated with the selected extract were observed by phase contrast microscopy using a Zeiss Axio Observer Z1 (Carl Zeiss AG, Jena, Germany) and an Operetta high-content imaging system (Perkin Elmer, Buckinghamshire, UK) for fluorescent microscopy. HeLa cells (2.5 × 10^4^ cell/mL, 0.1 mL/well) were incubated in a 96-well plate with 0.2 mL/well of three different doses (62.5, 125.0, and 250.0 µg/mL) of the selected extract of CN for 48 h at 37 °C with 5% CO_2_. The cells were directly imaged for phase contrast microscopy, whereas Hoechst 33,342 dye (Invitrogen, Paisley, UK; Cat. No.: H1399) was applied for fluorescent microscopy following the manufacturer’s instructions. The cells were stained with 0.5 μL dye (0.4 mg/mL) and incubated for 15 min prior to viewing. 

**Table 1:** IC_50_ (µg/mL) of CN Leaf Extracts on HeLa Cells

	IC_50_* (µg/mL)		
	Time of treatment (h)		
Extraction solvent	24	48	72
Hexane	200	185	170
DCM	100	70	80
Aqueous	> 4,000	1700	700
Crude 80% MeOH	1,300	650	650
Crude water	4,000	1,800	1,500

*IC_50_, half maximal inhibitory concentration

**Table 2:** Phytoconstituents Identified in the DCM Fraction of the CN Leaf Extract by GC-MS

Peak no.	Identified/Similar compounds	Molecular mass (g mol-1)	RT	Area (%)	Compound nature
1	Cyclohexane, isocyanato-	125.084	4.453	0.09	Hexahydrobenzene
2	Sulfuric acid, dimethyl ester	126.132	5.078	0.22	Diester
3	Naphthalene, 1,2-dihydro-1,1,6-trimethyl-	172.125	7.473	0.19	Aromatic hydrocarbon
4	Cyclodecane	140.27	8.16	0.18	Monocyclic alkane
5	Butylated hydroxytoluene	220.183	8.479	0.3	Phenol derivative
6	2-propenoic acid, 3-(3-hydroxyphenyl)-, methyl ester	178.063	9.285	0.08	Phenolic
7	1-Tridecene	182.203	9.389	0.16	Acyclic olefins; alkenes
8	4-((1E)-3-hydroxy-1-propenyl)-2-methoxyphenol	180.079	9.84	0.79	Phenolic
9	Pentadecanoic acid, 14-methyl-, methyl ester	270.256	10.722	2.48	Fatty acid
10	n-hexadecanoic acid	256.24	10.895	1.95	Fatty acid
11	Heptadecanoic acid, methyl ester	284.272	11.215	0.29	Fatty acid
12	Triphenylmethane	244.125	11.291	0.19	Hydrocarbon
13	1,3-dicyclohexylurea	224.35	11.499	0.19	Urea
14	9,12-octadecadienoic acid, methyl ester	294.256	11.569	1.22	Fatty acid
15	9,12,15-octadecatrienoic acid, methyl ester, (Z,Z,Z)-	292.24	11.603	3.34	Fatty acid
16	Phytol	296.308	11.652	1.15	Diterpene alcohol
17	Octadecanoic acid, methyl ester	298.287	11.694	1.12	Fatty acid
18	7,10,13-hexadecatrienoic acid, methyl ester	264.4	11.777	0.58	Fatty acid
19	Octadecanoic acid	284.484	11.853	0.72	Fatty acid
20	1-methyl-10,18-bisnorabieta-8,11,13-triene	256.219	12.048	0.15	Other organic compounds
21	1,2-benzenedicarboxylic acid, mono(2-ethylhexyl) ester	278.152	13.714	1.61	Ester derivative of benzoic acid
22	Hexadecane	226.44	13.915	0.42	Alkane hydrocarbon
23	N-(4-methoxyphenyl)-2-hydroxyimino-acetamide	194.069	14.436	4.72	Acetamide
24	9,12-octadecadienoic acid (Z,Z)-, 2-hydroxy-1-(hydroxymethyl)ethyl ester	354.277	14.589	3.16	Fatty acid
25	Nonanoic acid, 9-(3-hexenylidenecyclopropylidene)-, 2-hydroxy-1-(hydroxymethyl)ethyl ester, (Z,Z,Z)-cont.	352.261	14.658	2.74	Fatty acid
26	13-Docosenamide, (Z)-	337.334	15.248	2.98	Fatty acid amides
27	Vitamin E	430.381	19.476	0.64	Tocopherols and tocotrienols
28	Stigmasterol	412.702	21.94	0.58	Phytosterol (steroid derivative)

RT, Retention time

**Figure 2:**
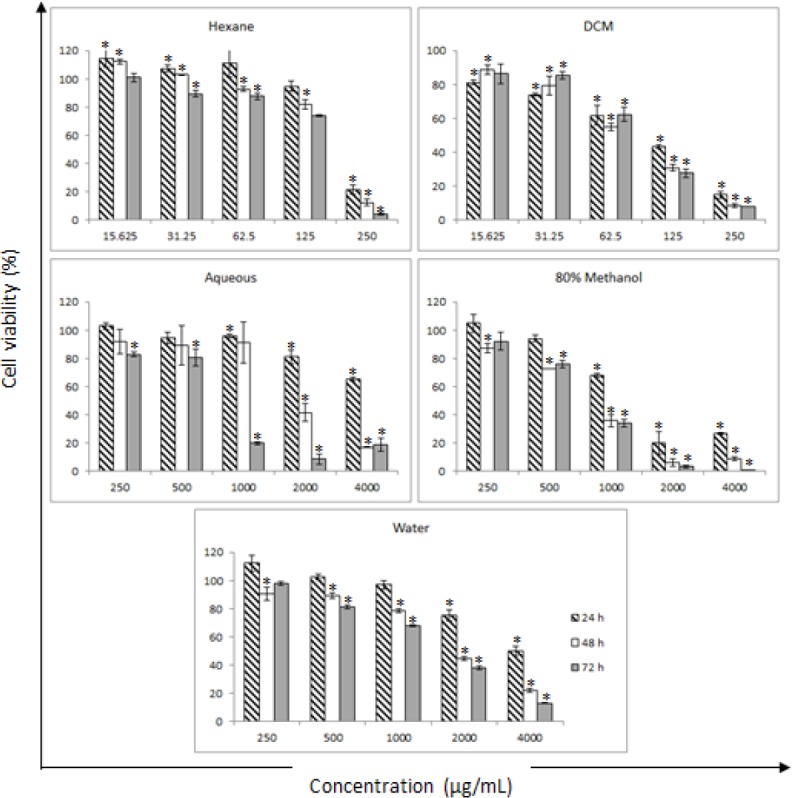
Antiproliferative Effects of CN Extracts on HeLa Cells. The cells were incubated with increasing concentrations of different solvent extracts (hexane, DCM, aqueous, 80% methanol, and water) for 24, 48, and 72 h, and cell viability was determined by the SRB assay. All values given are means ± SDs. Asterisks signify statistical significance at p < 0.05 compared to the untreated HeLa cells (100% viability)

**Figure 3:**
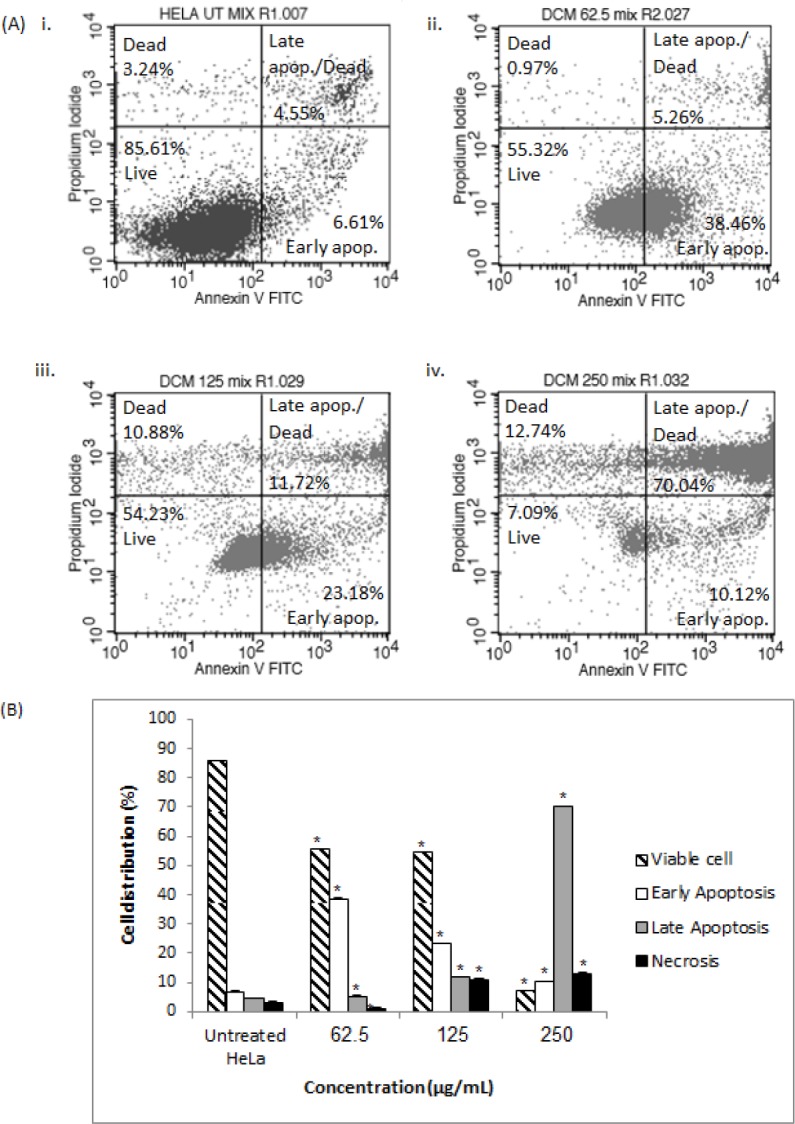
Dot Plots (A) of untreated HeLa cells (i) and HeLa cells (ii, iii, and iv) treated with 62.5, 125, and 250 µg/ml of the DCM fraction of the CN extract, respectively, and the percentage (%) of cell distribution (B) of HeLa cells after treatment with the DCM fraction as determined by Annexin V-FITC and PI staining. Asterisks signify statistical significance at p < 0.05 compared to untreated HeLa cells

**Figure 4:**
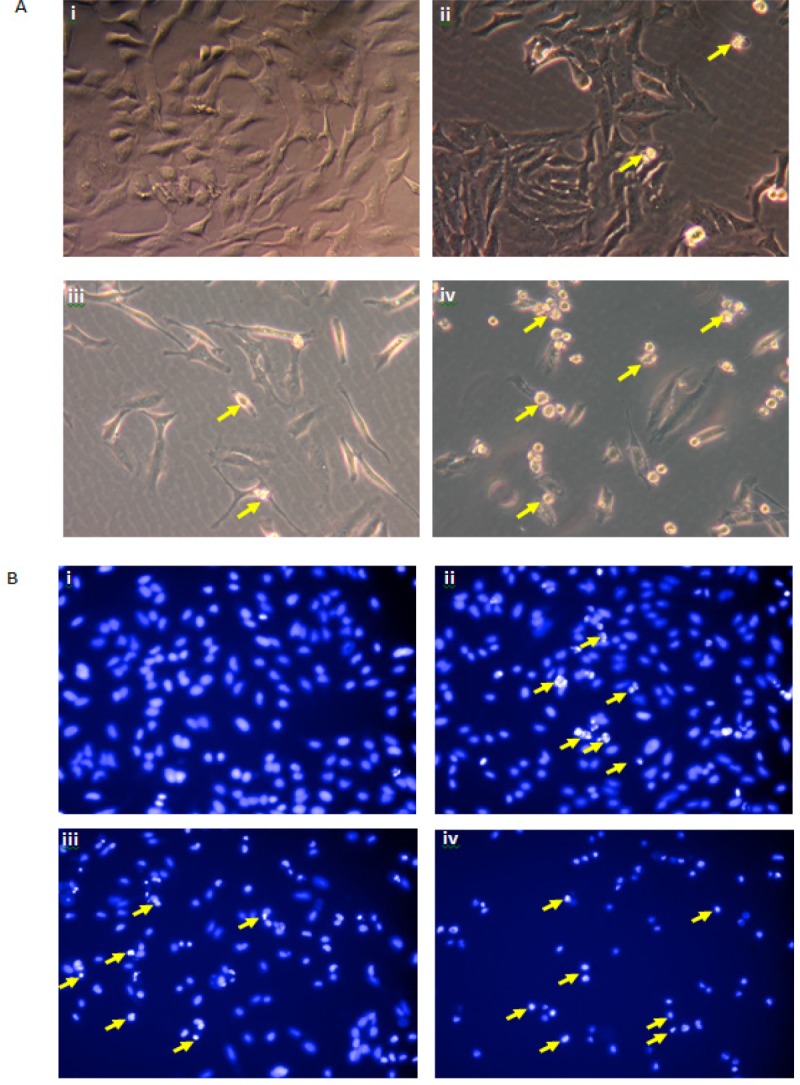
Representative Images Showing Morphological Changes of HeLa Cells Detected by (A) phase contrast microscope and (B) fluorescent microscope (Hoechst 33342): (i) Untreated HeLa cells, (ii, iii, iv) HeLa cells treated with the DCM fraction of the CN extract at 62.5, 125, and 250 µg/mL, respectively

**Figure 5:**
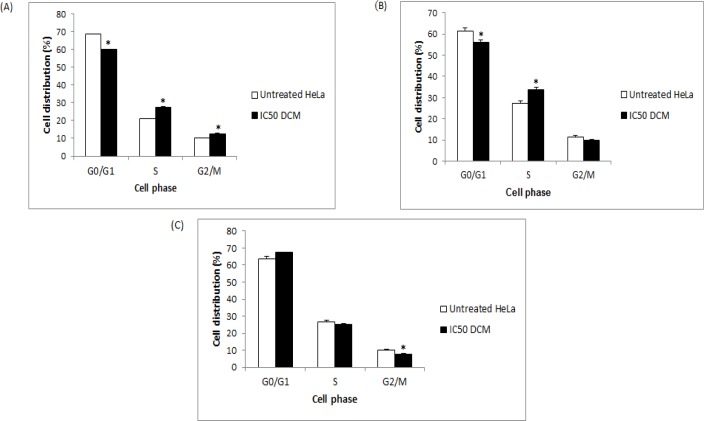
Effects of the DCM Fraction of the CN Leaf Extract on the Cell Cycle of HeLa Cells. The cells were treated at 70 µg/mL for (A) 24 h, (B) 48 h, and (C) 72 h. Asterisks signify statistical significance at p < 0.05 compared to untreated HeLa cells

*Cell cycle analysis*

HeLa cells (3.7 x 10^4^ cells/mL, 6 mL/flask) were incubated with the selected extract at the IC_50_ dose for 24, 48, and 72 h at 37°C with 5% CO_2_. The cells were then collected by trypsinization with Accutase (Nacalai Tesque, Kyoto, Japan; Cat. No.: 12679-54), washed in PBS, and fixed in 70% cold ethanol. Thereafter, the cells were stained using FxCycleTM PI/RNase staining solution (Life Technologies Corporation, Carlsbad, CA, USA; Cat. No.: F10797) following the manufacturer’s protocol. A FACS Calibur flow cytometer (BD Biosciences) was used to determine the distribution of cells in different stages of the cell cycle. The percentage of cells in the G1, S, and G2/M phases was calculated using ModFit LT software (Verity Software House, Topsham, Maine, USA).

*GC-MS analysis*

The selected CN extract was analyzed based on the optimized method using an Agilent 6890/5973 GC-MS system (Santa Clara, CA, USA). The separation was performed with an HP-5MS capillary column (30 m × 0.25 mm × 0.25 µm). The injector was set at 325°C (split mode with the ratio adjusted to 5:1, injection volume = 1 µL). The oven temperature started at 70°C for 2 min, increased to 280 °C, then was held for 20 min at 20°C ramp/min. The flow rate of the carrier gas (helium) was maintained at 1.2 mL/min. The mass spectrometer was operated with ionization in the electron impact mode using 70 eV (ionization energy) to obtain the mass spectra that ranged from m/z 40 to 700. Identifications were made by comparison with NIST library data.

*Statistical analysis*

The IC_50_ values of the antiproliferative activity of the extracts against HeLa cells were estimated from the dose-response curves. The experiments were repeated at least three times, with three samples for each repeat. The results are expressed as means ± standard deviation (SD). The data were analyzed using one-way analysis of variance to identify significant differences among groups. p < 0.05 was considered to be statistically significant. Analysis of the data was performed using SPSS statistical software version 22.0 (IBM Corporation, Armonk, NY, USA). 

## Results

*Antiproliferative activity of CN extracts against HeLa cells*

Table 1 presents the IC_50_ values of the CN extracts against HeLa cells, and Figure 2 shows the antiproliferative effects of the extracts. All extracts showed significant antiproliferative activity against HeLa cells in a dose- and time-dependent manner. The non-polar (hexane) and semi-polar (DCM) fractions had lower IC_50_ values than the other (polar) extracts. The DCM fraction had the highest toxicity against HeLa, with the lowest IC_50_ value (70 µg/mL) occurring at 48 h of treatment. Thus, treatment of HeLa cells with the DCM fraction for 48 h was selected for further analysis.

*Evaluation of apoptosis by flow cytometric analysis*

After treatment of HeLa cells with 62.5 µg/mL of the DCM fraction for 48 h, 55% of the cells were viable, 38% were in early apoptosis, 5% were in late apoptosis, and 1% exhibited necrosis (Figure 3). Treatment with 125 µg/mL of the DCM fraction increased the percentage of cells in late apoptosis (12%), and 70% of cells were in late apoptosis when treated with 250 µg/mL of the DCM fraction. The percentage of necrotic cells was less than 12% for all treatments.

*Morphological analysis*

HeLa cells treated with 62.5, 125.0, and 250.0 µg/mL of the DCM fraction of CN leaves showed reduced cell viability with increasing dose when compared with untreated cells ((Figure 4A(i)). Phase contrast microscopy showed that untreated HeLa cells were characterized by an organized monolayer with a regular polygonal appearance. After treatment, the cells became rounded and disconnected from each other and exhibited cellular shrinkage and nuclear condensation. Fluorescent microscopy with Hoechst 33,342 staining then was used to determine if the DCM fraction induced apoptosis in HeLa cells. Untreated HeLa cells remained uniformly stained blue ((Figure 4B(i)). Cells treated with 62.5 µg/mL of the extract showed some chromatin condensation and DNA fragmentation, which are the hallmarks of apoptotic cells. A number of cells with similar changes were observed in the 125 µg/mL treatment. At 250 µg/mL of the extract, most cells showed apoptotic body formation with decreased cell viability, indicating that induction of apoptosis was dose-dependent. 

*Cell cycle analysis*

The IC_50_ concentration of the DCM fraction of CN at 48 h (70 µg/mL) was used to evaluate the effects of the extract on the cell cycle of HeLa cells over time. A significant increase (p < 0.05) in HeLa cells in the S and G2/M phases compared to the control group was detected after 24 h of treatment (Figure 5A). This increase was accompanied by a significant decrease (p < 0.05) of HeLa cells in the G0/G1 phase. After 48 h, a significant increase (p < 0.05) in HeLa cells in the S phase only was detected, and it was accompanied by a decrease in cells in the G0/G1 phase (Figure 5B). After 72 h, a significant decrease (p < 0.05) in the population of cells in the G2/M phase was recorded (Figure 5C). 

*GC-MS analysis of the DCM fraction*

The GC-MS analysis of the DCM fraction of CN leaves revealed high flavonoid content. Identification of the compounds was made by referring to the corresponding compound in the NIST library with a similarity index of at least 80%. The biological potential of the identified compounds was determined by comparison with previously reported results. Table 2 shows the 28 phytochemical constituents identified in this study; the highest relative peak area was for N-(4-methoxyphenyl)-2-hydroxyimino-acetamide (4.72%). However, the identified phytochemicals belonged mainly to the fatty acid class, as it covered 20.29% of the total peak area.

## Discussion

None of the CN extracts tested in this study were cytotoxic to HeLa cells as defined by the American National Cancer Institute’s (IC_50_: < 30 μg/mL) criteria of cytotoxic activity of crude extracts. Of the extracts tested, the DCM fraction had the lowest IC_50_ value. This finding is in agreement with results of previous studies (Arullappan et al., 2014; Che Sulaiman et al., 2015; Ng et al., 2017; Yong et al., 2013) in which other non-polar and semi-polar extracts of CN, such as chloroform, ethyl acetate, hexane, and petroleum ether, demonstrated higher antiproliferative activity than polar extracts (aqueous and methanol). This study was the first to report this result for the DCM fraction. 

Flow cytometry data showed that the DCM fraction at 48 h affected the progression of apoptosis in a dose-dependent manner, whereby the lowest dose (62.5 µg/mL) induced the highest percentage of early apoptosis and the highest dose (250 µg/mL) resulted in the most cells in the late apoptosis stage. A previous study showed that cold aqueous and crude methanol CN leaf extracts induced apoptosis in a wild type BRAF melanoma cell line (D24) in a dose- and time-dependent manner (Fong, 2015). In another study, mouse hepatoma cells treated with 30% ethanol extracts of CN in low or high doses showed a significant decrease in the expression of PCNA and p-AKT (proliferation markers) and increased expression of BAX, Bcl2, caspase-3, and PAPR (apoptosis markers) (Huang et al., 2015). Ng et al., (2017) reported that high concentration (> 100 µg/mL) of hexane extract from CN induced apoptosis in A549, CNE1, and HepG2 cell lines by upregulating activities of caspase 3/7, 8, and 9. Morphological changes of treated HeLa cells, such as cell shrinkage, apoptotic body formation, and DNA fragmentation, also were indicative of apoptosis.

Cell cycle analysis showed that the DCM fraction induced S and G2/M phase arrest after 24 h of treatment and S-phase only after 48 h of treatment. DNA synthesis or duplication of chromosome occurs during S-phase, whereas the G2/M phase is a gap state (G2) in which the cell with newly replicated DNA continues to grow to ensure that everything is ready to enter the mitosis (M) phase. At the M stage, the cell stops growing and divides its copied DNA into two daughter cells. The observed S phase cell cycle arrest, which indicates decreasing rate of DNA synthesis, might explain the mechanism of the antiproliferative effect by the DCM fraction at 48 h of treatment. In a previous study, the hexane fraction of CN increased the sub-G1 populations of CNE1, HepG2, and A549 cells in a dose-related manner, while the G0/G1 and G2/M populations showed a corresponding decrease (Ng et al., 2017). Thus, the antiproliferative mechanism may differ depending on the type of cancer cell, the tested compound, and the different natural products present in the extract, which are likely to vary at different stages of the cell cycle (Peng et al., 2015). 

Twenty-eight volatile components of the DCM fraction were identified via GC-MS analysis, and fatty acids were the main components. Some of the same phytoconstituents were reported in other GC-MS studies of CN, including 1,2-benzene dicarboxylic acid mono(2-ethylhexyl) ester, 9,12,15-octadecatrienoic acid methyl ester (Z,Z,Z)-, n-hexadecanoic acid, octadecanoic acid methyl ester, 9,12-octadecadienoic acid (Z,Z)-, phytol, stigmasterol, and vitamin E (Abdul Rahim et al., 2016; Alam et al., 2017; Mustapa et al., 2015; Ng et al., 2017; Teoh et al., 2017; Yong et al., 2013). Some of the compounds identified in this study were previously reported to have antiproliferative (cytotoxic and antioxidant) activity against tumor cell lines, such as 1,2-benzenedicarboxylic acid mono(2-ethylhexyl) ester (Krishnan et al., 2014), 4-((1E)-3-hydroxy-1-propenyl)-2-methoxyphenol (Duke, 1992), phytol (Lakshmi et al., 2016; Santos et al., 2013, Song and Cho, 2015), stigmasterol (Gao et al., 2008; Huang et al., 2009), and vitamin E (Campbell et al., 2006; Howard et al., 2011). 

Further studies are needed to discover the mechanisms underlying the antiproliferative activity of *C. nutans* extracts against cancer cell lines and to identify specific bioactive compounds that are responsible for the observed activities. This study shows that different solvent extracts of CN can have different antiproliferative activities against HeLa cells. Moreover, the extraction technique and fraction used can affect the outcome, as the DCM fraction in this study had a lower IC_50_ value against HeLa cells (70 µg/mL) than has been reported in previous studies. The potent phytochemical constituents of the DCM fraction may contribute to the antiproliferative activity against HeLa cells, and this information may be useful for the development of a novel anticancer therapy. 

## Funding Statement

This work was funded and supported by a FRGS grant (203/CIPPT/6711340) from the Ministry of Higher Education, Government of Malaysia and USM Bridging Grant (304/CIPPT/6316239) to Hasni Arsad.
